# Multiscale experimental study on the effects of different weight-bearing levels during moderate treadmill exercise on bone quality in growing female rats

**DOI:** 10.1186/s12938-019-0654-1

**Published:** 2019-03-22

**Authors:** Juan Fang, Jiazi Gao, He Gong, Tianlong Zhang, Rui Zhang, Bangchao Zhan

**Affiliations:** 1 Department of Engineering Mechanics, Jilin University, Changchun, 130022 People’s Republic of China; 2 School of Civil Engineering, Jilin Jianzhu University, Changchun, 130118 People’s Republic of China; 3 School of Biomedical Science and Medical Engineering, Beihang Univerisity, Beijing, 100191 People’s Republic of China

**Keywords:** Weight-bearing, Moderate intensity, Treadmill exercise, Bone quality

## Abstract

**Background:**

Bone tissue displays a hierarchical organization. Mechanical environments influence bone mass and structure. This study aimed to explore the effects of different mechanical stimuli on growing bone properties at macro–micro–nano scales.

**Methods:**

Sixty five-week-old female Wistar rats were treadmill exercised at moderate intensity with the speed of 12 m/min, and then randomly divided into five groups according to weight-bearing level. After 8 weeks of experiment, femurs were harvested to perform multiscale tests.

**Results:**

Bone formation was significantly increased by weight-bearing exercise, whereas bone resorption was not significantly inhibited. Trabecular and cortical bone mineral densities showed no significant increase by weight-bearing exercise. The microstructure of trabecular bone was significantly improved by 12% weight-bearing exercise. However, similar positive effects were not observed with further increase in weight-bearing levels. The nanomechanical properties of trabecular bone were not significantly changed by weight-bearing exercise. The macrostrength of whole femur and the nanomechanical properties of cortical bone significantly decreased in the 19% and 26% weight-bearing exercise groups.

**Conclusion:**

When rats ran on the treadmill at moderate intensity during growth period, additional 12% weight-bearing level could significantly increase bone formation, improve microstructure of trabecular bone, as well as maintain the structure and mechanical properties of cortical bone. Excessive weight-bearing level caused no positive effects on the trabecular bone microstructure and properties of cortical bone at all scales. In addition, increased weight-bearing level exerted no significant influence on trabecular and cortical bone mineral densities.

## Background

Running is one of the most common weight-bearing exercises and it can increase bone mass in humans and animals [1–5]. Numerous experiments show that the effect of treadmill exercise on bone mass is related to exercise intensity and the effect of the moderate-intensity treadmill exercise on increasing bone mass and strength is better than those of low- and high-intensity treadmill exercise [3–10]. Thus, the moderate-intensity treadmill exercise may be the most suitable form within these three exercise intensity levels.

The effect of treadmill exercise on bone mass is associated with the peak strain on the bone produced by exercise; and animal studies have reported that once the mechanical strain induced by exercise exceeds a certain threshold, bone formation is positively correlated with the peak strain magnitude [10–12]. Treadmill running may increase more bone mass in the long bones of rats at the main weight-bearing sites than the less weight-bearing sites [13]. Increasing the weight of backpack can effectively increase the peak strain, thus it can further effectively increase bone mass [14–19]. A previous study reports that moderate-intensity treadmill exercise with a 19–20% body weight backpack can significantly increase bone mineral content in the hind limbs of 5-month-old female rats [18]. This form of exercise also significantly increases the bone mineral density (BMD) and bone mineral content of trabecular bone in 3-month-old ovariectomized female rats [19]. Accordingly, moderate treadmill exercise in combination with a certain additional load in backpack may effectively increase bone mass in adult rats.

The responses of bone to mechanical stimuli are distinct at different growth stages, and the sensitivity of bone to mechanical loading is significantly higher in young mice than in adult and aged animals [20]. Treadmill exercise during the growth period has positive effective on bone quality [1, 14, 15, 21–25]. Most of the subjects in previous studies are adult rats, and only one weight-bearing level is established. The effect of treadmill exercise with different weight-bearing levels on bone quality in growing rats remains unclear. Our previous study investigated the effects of treadmill exercise with different weight-bearing levels on the subchondral trabecular bone microstructure and articular cartilage morphology of the knee joint in growing female rats [26]. Results showed that the effect of treadmill exercise on articular cartilage is associated with weight-bearing levels. The microstructure of subchondral trabecular bone is significantly improved by 12% weight-bearing level. Nevertheless, the bone mineral density (BMD) and bone volume fraction (BV/TV) are decreased, and the structure model index (SMI) is increased by 26% weight-bearing level; thus, this exercise exerts no positive effects on the microstructure of trabecular bone [26]. However, in our previous study, the influence of treadmill exercise with weight-bearing is only investigated on articular cartilage in growing female rats. The effect of this form of mechanical load on bone quality remains unknown.

Accordingly, moderate treadmill exercises with different weight-bearing levels groups were established in this study. Bone displays hierarchical structural organization and has different properties at macro–micro–nano scales [27]. Bone properties may be affected by treadmill exercise from macro to nano scale. The aim of this study was to explore the effect of different weight-bearing levels during moderate treadmill exercise on bone quality (i.e. BMD, bone geometry, microstructure, macromechanical and nanomechanical properties) in growing female rats at macro–micro–nano scales.

## Methods

### Experimental design

This study was performed in strict accordance with the recommendations of the Laboratory Animal Standardization Committee. The protocol was approved by the Medical Ethics Committee of the No. 1 Hospital of Jilin University (No. 2013-145).

**Fig. 1:**
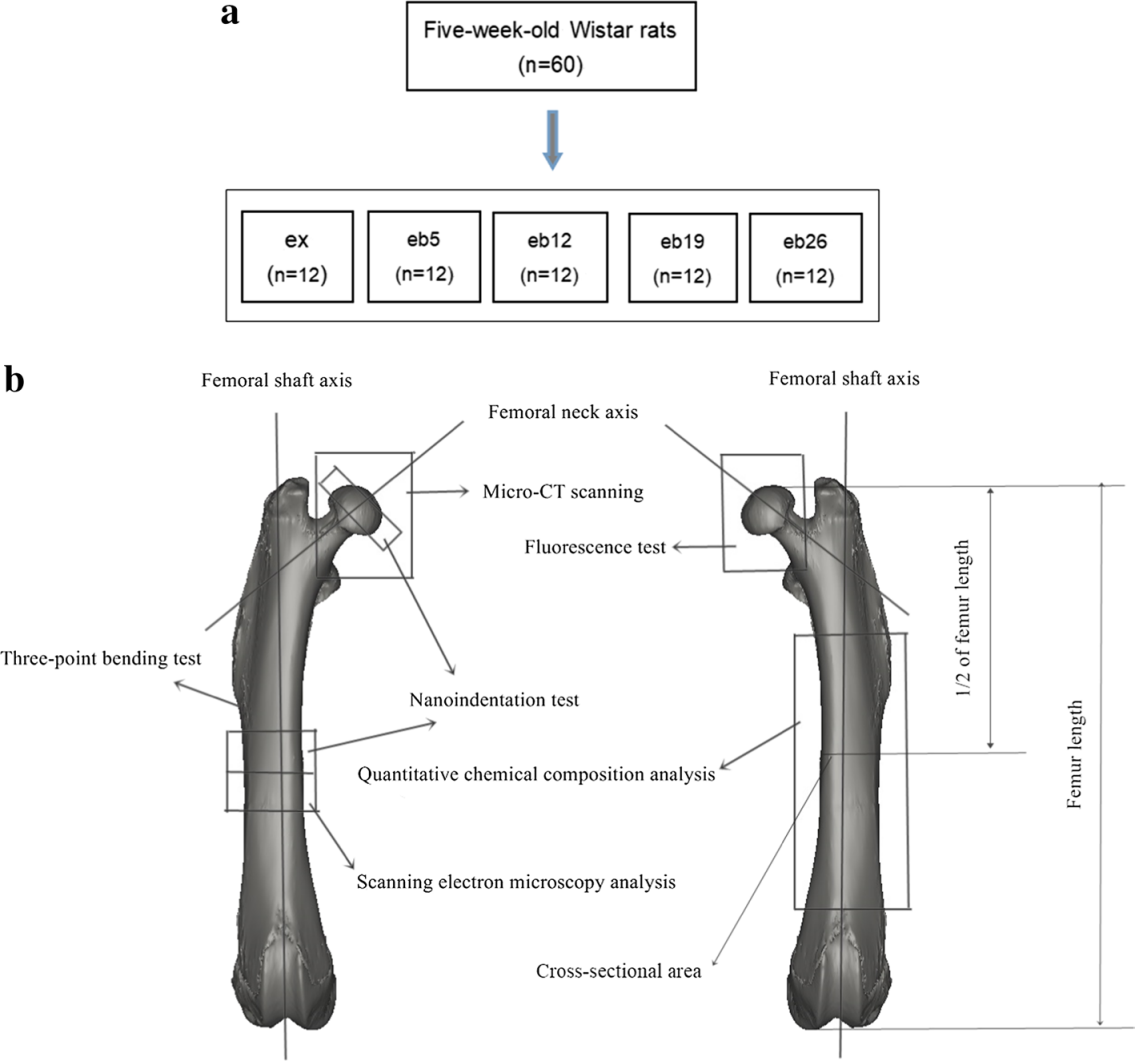
The schematic of experimental grouping and the measurements performed in the femur. **a** Experimental grouping. **b** Measurement of the femur

A total of 60 5-week-old female Wistar rats were purchased from the Experimental Animal Center of Jilin University. All rats with the body weight 123.07 ± 16.33 g arrived at the laboratory a week before the experiment began to accommodate the diet and new environment. These rats were provided with standard rodent diet (autoclaved NIH-3 with 6% fat; 18% protein; Ca:P, 1:1; and fortified with vitamins and minerals) and tap water during the experimental period. The environmental temperature was 24 ± 2 °C under natural light conditions. All rats were randomly divided into five groups with 12 animals each. The schematic of experimental grouping is shown in Fig. 1a. Weight-bearing was in the form of backpack; the backpack weights were 5%, 12%, 19%, and 26% of the individual body weight of rats. The five groups are as follows: treadmill exercise control group (ex), treadmill exercise with 5% weight-bearing level group (eb5), treadmill exercise with 12% weight-bearing level group (eb12), treadmill exercise with 19% weight-bearing level group (eb19), and treadmill exercise with 26% weight-bearing level group (eb26).

Our previous study demonstrates that, according to blood lactate accumulation, for 5-week-old female rats, treadmill exercise at 12 m/min for 15 min correspond to moderate intensity exercise [26]. Therefore, all rats in this experiment ran at 12 m/min for 15 min in each day.

### Exercise protocol

All rats exercised 7 days/week for 8 weeks. During the first 2 weeks, the rats were conditioned to treadmill exercise and wore backpack. In this period, velocity was gradually increased from 8 m/min to 12 m/min with the increment of 3% per day for 15 min/day and 7 days/week. The backpack weight of rats in all weight-bearing groups was gradually increased from 0 to the target weight, with the increment of 7% of the target weight borne by the rats in each group per day. The duration of treadmill exercise with weight-bearing groups was 15 min/day, and no weights at other time points. The form of backpack is consistent with that used in the literature, and the backpack is filled with leaden strips [18]. The body weight of each rats were measured once a week. The load in the backpack was adjusted to the change in body weight.

### Specimens preparation

At 13 days before killing, all rats were treated with the subcutaneous injection of calcein (5 mg/kg). At 3 days before killing, calcein were re-injected to create a fluorescence marker [23, 28–30]. After 8 weeks of experiment, the rats were killed by collecting blood samples from the abdominal aorta under pentobarbital sodium anesthesia, and the left lower limb calf muscles (gastrocnemius and soleus muscles) of each animal were removed and weighed immediately. Bilateral femurs were prepared for testing, following removal of skin, muscles, and tendons. The length of left femurs was measured from femoral head to the distal medial condyle (Fig. 1b). The maximum and minimum external diameters positioned at 1/2 of the total left femur were measured (Fig. 1b), and the cross-sectional area was calculated. Schematic of the measurements performed in this study is shown in Fig. 1b.

### Serum test

Not less than 5 ml of blood was collected from the abdominal aorta under general anesthesia and centrifuged at 3000 rpm/min for 15 min to obtain serum samples. The alkaline phosphatase (ALP) and tartrate-resistant acid phosphatase (TRACP) in the serum were marked by enzyme linked immunosorbent assay (ELISA kit, Nanjing, China) using a Microplate reader (ELx800, BioTek Corporation, USA). Afterward, the rates of bone formation and resorption were determined.

### Three-point bending mechanical test

The right femurs were cleaned and kept wet during test by normal saline. Three-point bending mechanical test was conducted with the right femur loaded at mid-length in the anterior–posterior direction using an electronic universal testing machine (AG–X plus, Shimadzu, Japan). The test was continued until fracture, with a fulcrum span of 20 mm and an actuator velocity of 1 mm/min. Deflection (mm) and failure load (N) were obtained from load–displacement curve. Energy absorption (J) was defined by integrating the force–displacement curve from zero to the maximum force. Stiffness (N/mm) was determined from the slope of the linear portion (10–60% maximum force) of the load–displacement curve. Macro-elastic modulus (*E*) of the femur is calculated using the following equation:

1 $$E = Fl^{3} /48If$$

where *F* is failure load, *l* is span, *f* is deflection, and *I* is moment of inertia of fracture surface caused by three-point bending mechanical test, which is calculated from the micro-CT image.

### Histomorphometry

After three-point bending mechanical test, the right proximal femurs were fixed with 70% concentration ethanol and dehydrated progressively in 70%, 80%, 90%, and 100% EtOH (24–48 h per phase), and all proximal femur samples were embedded separately in polymethylmethacrylate. The embedded samples were cut along the coronal plane with a low-velocity diamond saw under constant deionized water irrigation, from which the proximal trabecular bone was exposed. For histomorphometry, 5-mm thick slices were cut along the coronal plane with a thin-sliced cutting machine for hard materials.

Laser scanning confocal microscopy (FV500, Olympus Corporation, Japan) was used to obtain images of right proximal femur samples, and one image per right femur sample was obtained. The mineral apposition rate (MAR), and the ratio of mineralizing surface to bone surface (MS/BS) of trabecular bone of 1 mm^2^ region of interest (ROI) in the right femoral head were calculated according to the standard nomenclature described by Parfitt et al. measured by Image-Pro Plus software [31, 32].

### Micro-CT scanning

The microarchitecture of the left femur was scanned by a micro-CT system (Skyscan 1076, Skyscan, Belgium), and then quantitative analysis of the 3D microarchitectures of trabecular bone in the femoral head and cortical bone in the femoral shaft was performed. The spatial resolution for specimen scanning was 18 μm. The scanned data was reconstructed by using NRecon software (NRecon, Skyscan, Belgium). BV/TV, Tb.Th, Tb.N, Tb.Sp, and BMD of trabecular bone (Tb.BMD) in the femoral head, and BMD, and thickness of cortical bone (i.e. Ct.BMD, Ct.Th) in the femoral shaft were calculated by CTAn (CTAn, Skyscan, Belgium).

The ROI in the micro-CT images was selected manually. The ROI for femoral head was all trabecular bone within the femoral head, and trabecular bone was selected as much as possible in the ROI. The ROI for the femoral shaft was positioned at 1/2 of the total left femur with 2 mm-thick from distal to proximal (Fig. 1b) [33], and 106 2D micro-CT images of each sample were selected.

### Nanoindentation test

After micro-CT scanning, longitudinal trabecular bone samples with a thickness of 2 mm cut from the left femoral heads along the axis of femoral neck (Fig. 1b) were used for nanoindentation test, and then the longitudinal indentation modulus (*E*_*Tb.L*_) and longitudinal hardness (*H*_*Tb.L*_) of trabecula were measured. Longitudinal cortical bone samples with a thickness of 2 mm cut from the femoral shaft along the axis of the femoral shaft were used for nanoindentation test, and then the longitudinal indentation modulus (*E*_*Ct.L*_) and longitudinal hardness (*H*_*Ct.L*_) of cortical bone were measured. The femoral shaft was cut along its axis to expose the transverse section of cortical bone, and transverse cortical bone samples were obtained for nanoindentation test; the transverse indentation modulus (*E*_*Ct.T*_) and transverse hardness (*H*_*Ct.T*_) of cortical bone were measured. All samples were dehydrated similar to the right femur, and the dehydrated samples were embedded in epoxy resin [34]. All embedded samples were metallographically polished using silicon carbide abrasive papers of decreasing grit sizes (400, 800, 1500, 2000, 3000, and 5000 grit), and finally on microcloths with finer grades of diamond suspensions to the finest, 0.05 μm grit, to produce smooth surfaces for nanoindentation test. The specimens were washed in deionized water between each polishing step to remove debris.

Nanoindentation tests were performed using Nano Indenter G200 (Nano G200, Agilent Technologies, Ltd., USA) with a Berkovich diamond indenter. The test was performed by driving the indenter into the specimen to a depth of 1000 nm at a constant loading rate of 750 μN/s (held for 10 s) and then unloading to 15% of the peak load at a rate equal to half that used during loading [23].

**Fig. 2:**
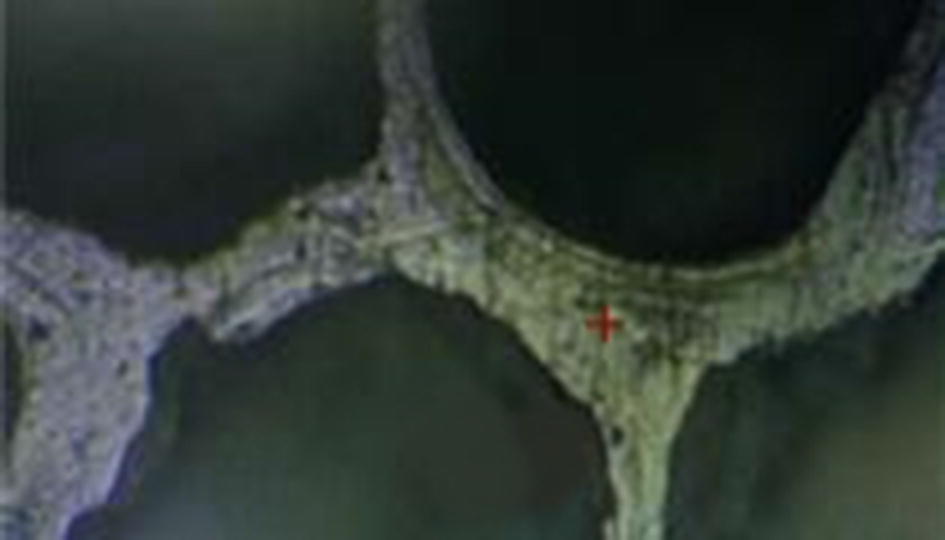
Indentation sites for nanoindentation test. Actual indented sites were marked by red cross under optical microscopy, and the sample thickness was 2 mm

For trabecular bone samples, all indents were conducted at the similar site based on the optical microscopy observation to eliminate any local effects (Fig. 2), 5 indentations were made in each sample, and then the mean value was calculated, as well as each cortical specimen. The indentation modulus (*E*) and hardness (*H*) were determined using the method of Oliver and Pharr [35].

*H* is calculated by the following equation:

2 $$H = P_{\hbox{max} } /A$$

where *P*_*max*_ is peak load, and *A* is contact projected area of the Berkovich indenter tip.

The relationship between effective modulus for the indenter-specimen combination (*E*_*ef*_) and slope (*S*) of the linear stage in the upper part of the unloading curve is:

3 $$S = 2\pi^{ - 1/2} \beta E_{ef} A^{1/2}$$

Effective modulus for the indenter-specimen combination *E*_*ef*_ can be derived from:

4 $$1/E_{ef} = (1 - \nu_{b}^{2} )/E_{b} + (1 - \nu_{i}^{2} )/E_{i}$$

where *ν* and *E* are Poisson’s ratio and elastic modulus of bone, respectively. The subscripts *b* and *i* represent bone and diamond indenter, respectively. *β* is indenter constant. Poisson’s ratio of bone *ν*_*b*_= 0.3; Poisson’s ratio (*ν*_*i*_) and elastic modulus (*E*_*i*_) of the Berkovich diamond indenter are 0.07 and 1140 GPa, respectively. Indenter constant *β *= 1.034 [23].

### Statistical analysis

One-way ANOVA was used to test the main effect of moderate treadmill exercise with different levels weight-bearing [36]. Data analysis was performed with SPSS 19.0 software, and the significance level was set to p < 0.05.

## Results

**Fig. 3:**
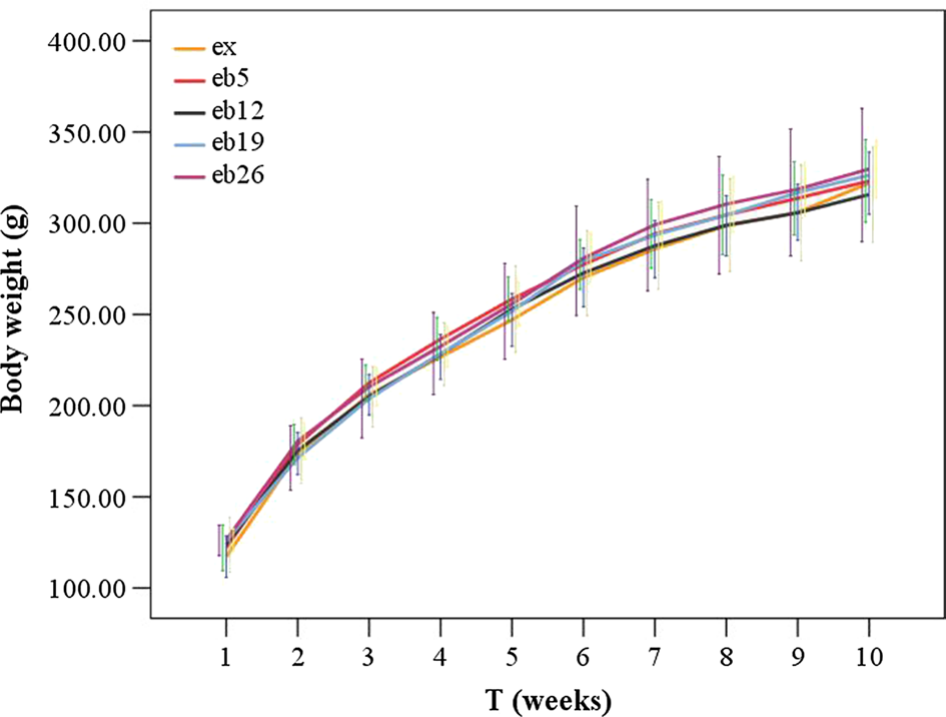
The body weight of each group during the experiment. Error bars represent standard deviation (SD). At T2, the 8-week exercise program started

**Fig. 4:**
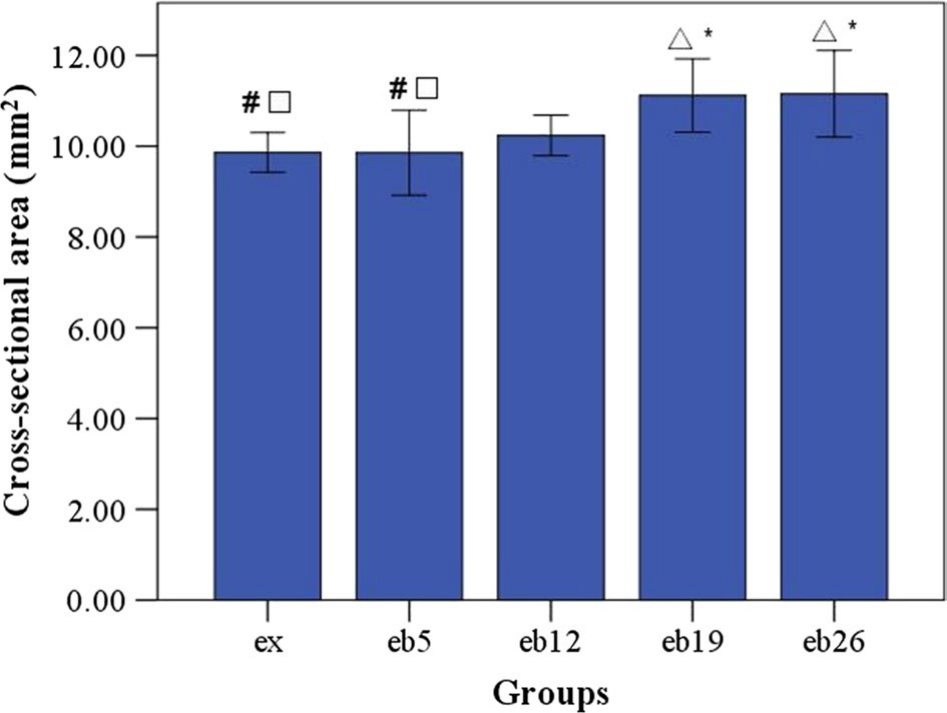
The cross-sectional areas in the middle segment of femoral shaft at the end of experiment. Error bars represent SD. ^△^Statistically different from the ex group (p < 0.05); *Statistically different from the eb5 group (p < 0.05); ^◇^Statistically different from the eb12 group (p < 0.05); ^#^Statistically different from the eb19 group (p < 0.05); ^□^Statistically different from the eb26 group (p < 0.05)

**Fig. 5:**
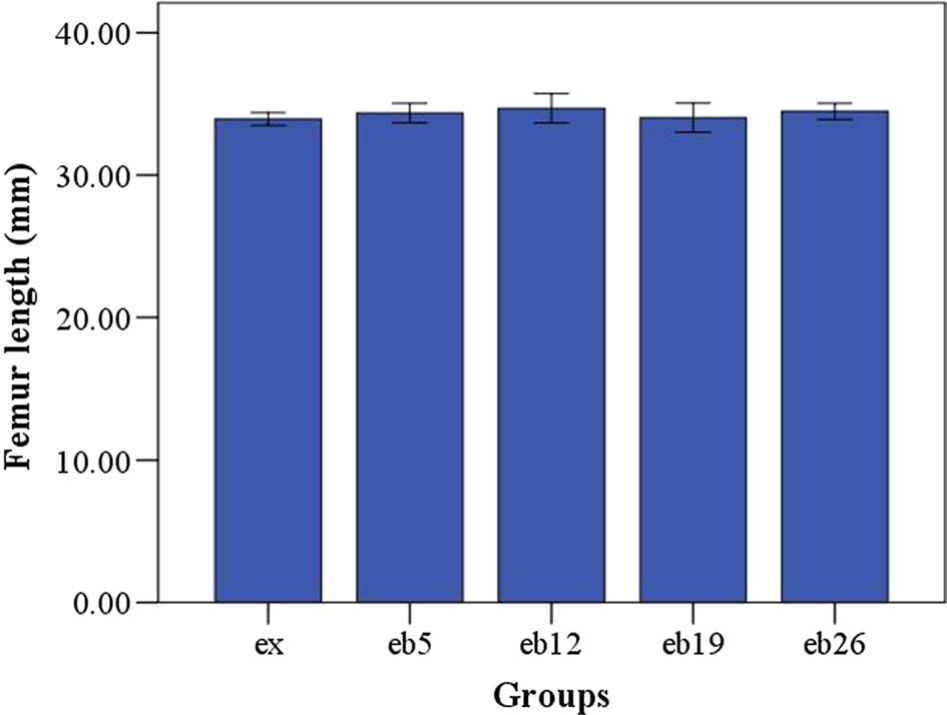
The length of femur. Error bars represent SD

**Fig. 6:**
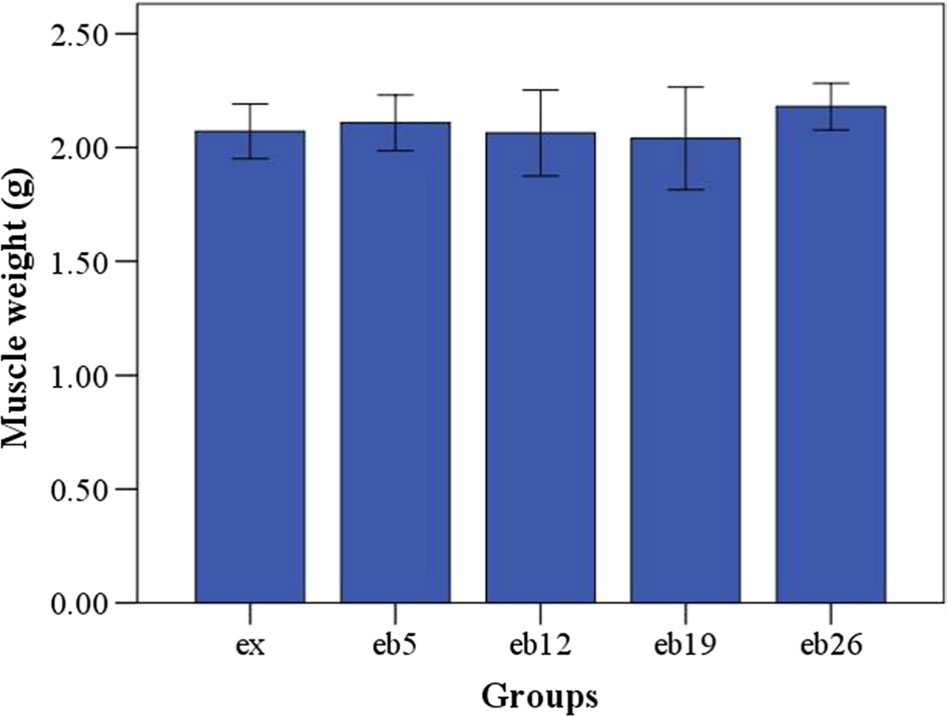
The calf muscles weight. Error bars represent SD

Figure 3 shows the body weight of each rat group during the experiment. Increasing weight-bearing level showed no significant effects on body weight for all time points (p > 0.05). The cross-sectional area in the middle segment of femoral shaft, length of the left femur, and calf muscles weight of rats in each group are shown in Figs. 4, 5, 6, respectively. No significant differences in femur length and calf muscles weight were detected among groups (p > 0.05, Figs. 5, 6).

### Serum test

**Fig. 7:**
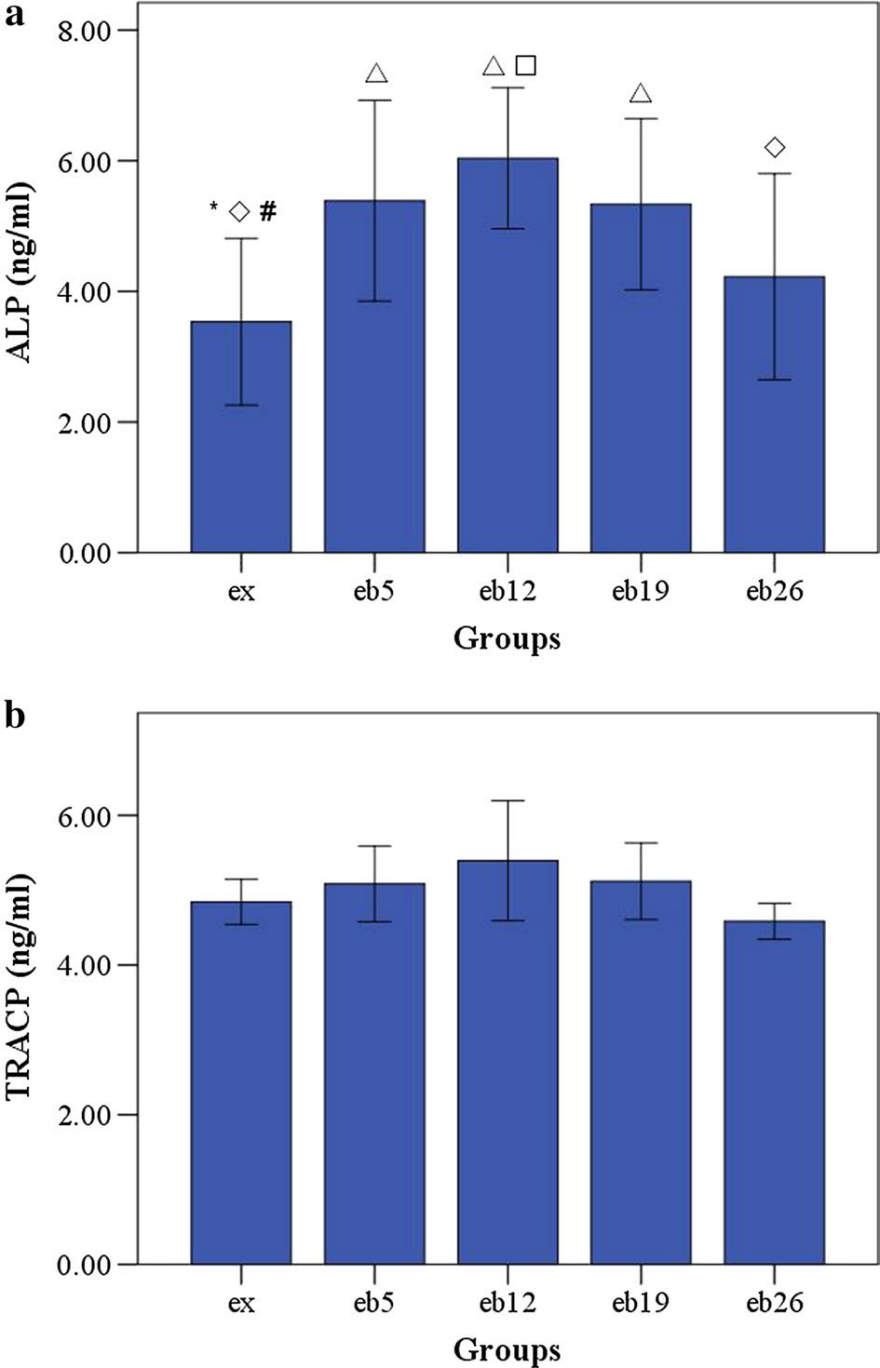
The serum concentrations of ALP and TRACP in each group. Error bars represent SD. ^△^Statistically different from the ex group (p < 0.05); *Statistically different from the eb5 group (p < 0.05); ^◇^Statistically different from the eb12 group (p < 0.05); ^#^Statistically different from the eb19 group (p < 0.05); ^□^Statistically different from the eb26 group (p < 0.05). **a** ALP concentration. **b** TRACP concentration

Figure 7 presents the serum concentrations of ALP and TRACP in each group. The ALP concentrations in the eb5, eb12, and eb19 groups were significantly higher than that in the ex group (p < 0.05); the ALP concentration in the eb12 group was the highest. The serum TRACP concentrations showed no significant differences among the groups (p > 0.05).

### Three-point bending mechanical test

**Fig. 8:**
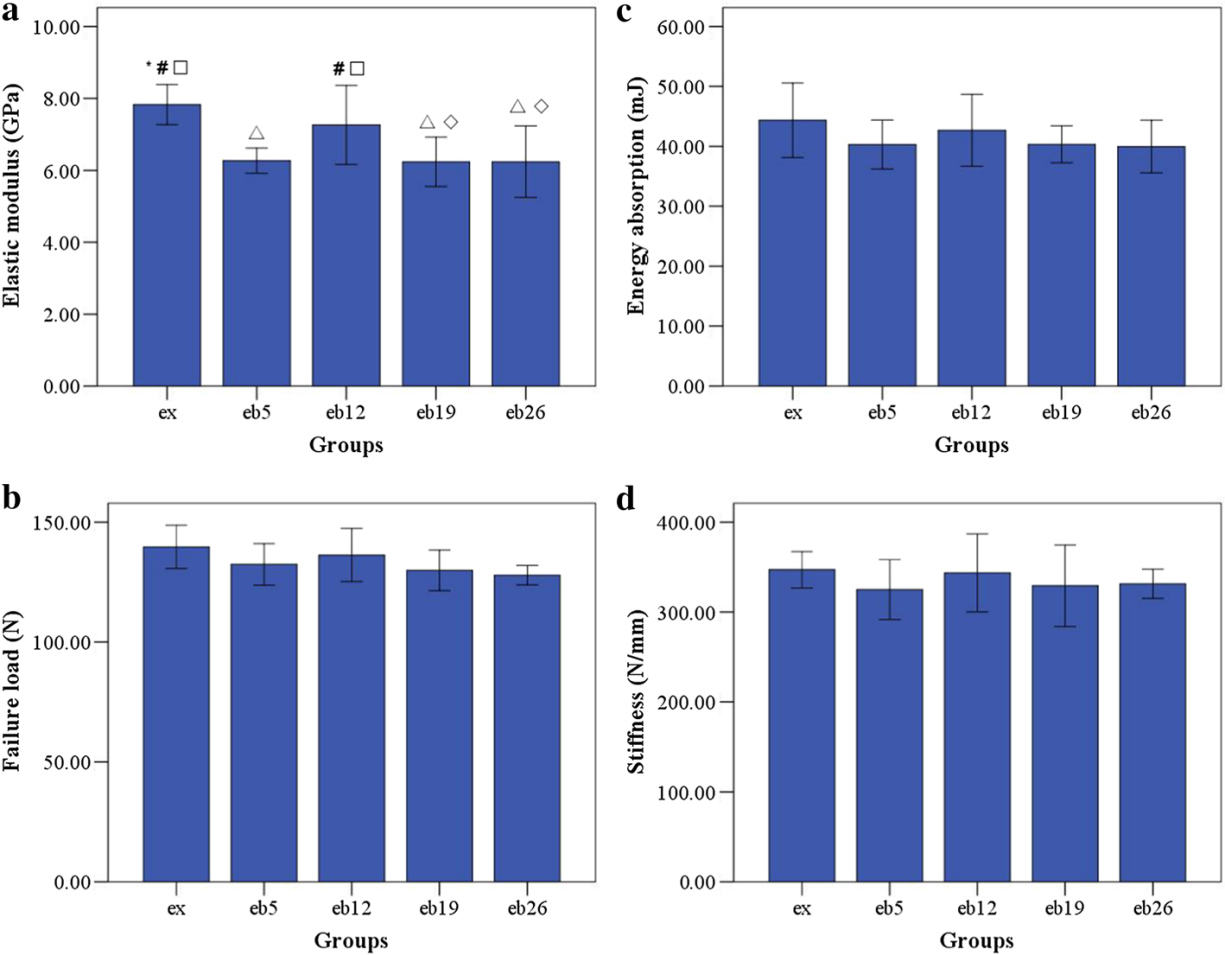
The macromechanical parameters of the left femur measured by three-point bending mechanical test. Error bars represent SD. ^△^Statistically different from the ex group (p < 0.05); *Statistically different from the eb5 group (p < 0.05); ^◇^Statistically different from the eb12 group (p < 0.05); ^#^Statistically different from the eb19 group (p < 0.05); ^□^ Statistically different from the eb26 group (p < 0.05). **a** Elastic modulus. **b** Failure load. **c** Energy absorption. **d** Stiffness

The macromechanical parameters of the left femur are shown in Fig. 8. The elastic moduli in the eb5, eb19, and eb26 groups were significantly lower than that in the ex group (p < 0.05). Nonetheless, no significant differences in the failure load, energy absorption and stiffness were observed among the groups (p > 0.05).

### Histomorphometry

**Fig. 9:**
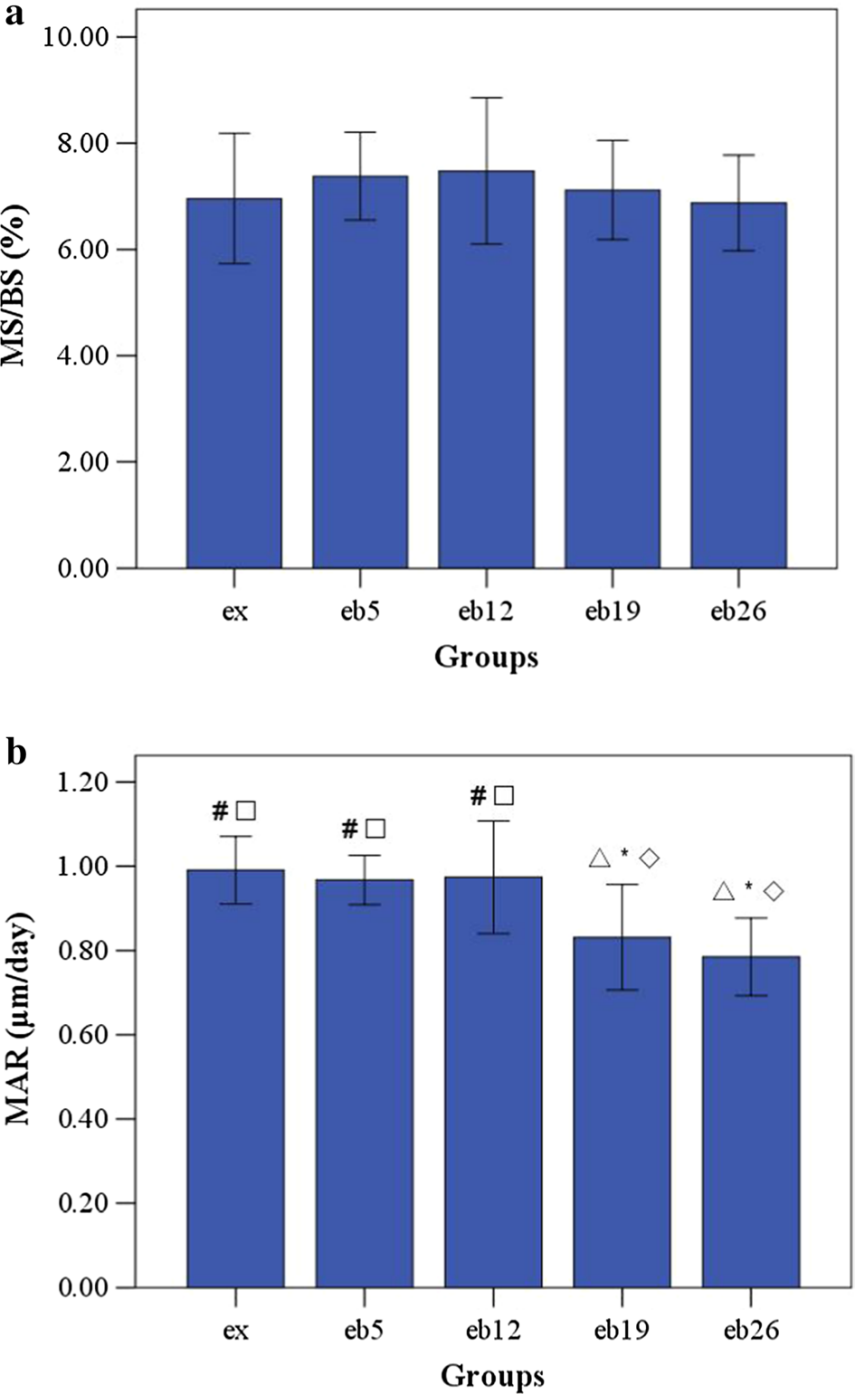
The MS/BS and MAR of trabecular bone in the femoral head. Error bars represent SD. *MAR* mineral apposition rate, *MS/BS* the ratio of mineralizing surface to bone surface. ^△^Statistically different from the ex group (p < 0.05); *Statistically different from the eb5 group (p < 0.05); ^◇^Statistically different from the eb12 group (p < 0.05); ^#^Statistically different from the eb19 group (p < 0.05); ^□^Statistically different from the eb26 group (p < 0.05). **a** MS/BS, **b** MAR

The MAR and MS/BS of trabecular bone are shown in Fig. 9. The MAR values in the eb19 and eb26 groups were significantly lower than those in the ex, eb5, and eb12 groups (p < 0.05). No significant differences were observed in the MS/BS among the groups (p > 0.05).

### Micro-CT scanning

**Fig. 10:**
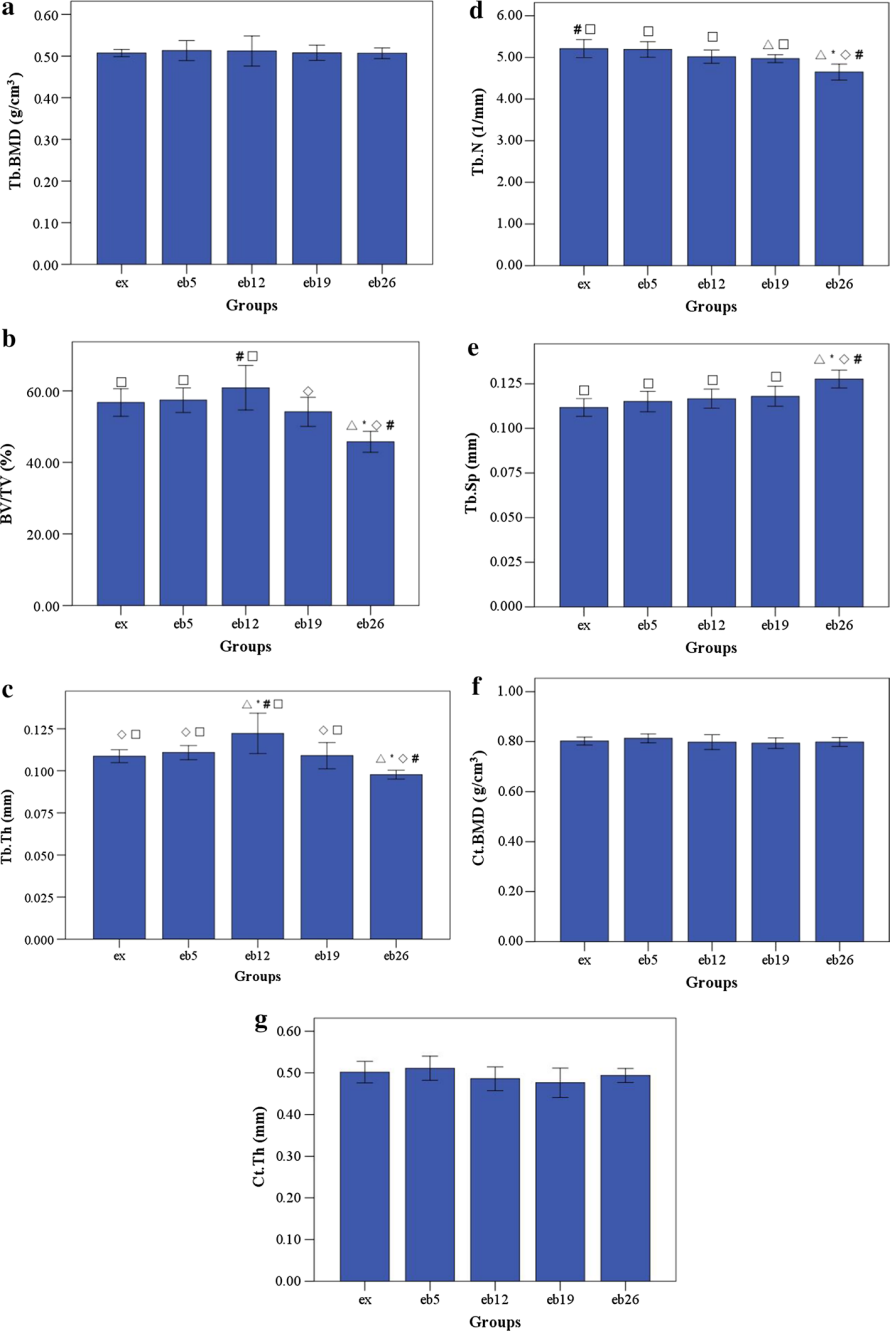
The 3D microstructure parameters of trabecular bone in femoral head and cortical bone in femoral shaft. Error bars represent SD. *Tb.BMD* trabecular bone mineral density, *BV/TV* bone volume fraction, *Tb.Th* trabecular thickness, *Tb.N* trabecular number, *Tb.Sp* trabecular separation, *Ct.BMD* cortical bone mineral density, *Ct.Th* cortical bone thickness. ^△^Statistically different from the ex group (p < 0.05); *Statistically different from the eb5 group (p < 0.05); ^◇^Statistically different from the eb12 group (p < 0.05); ^#^Statistically different from the eb19 group (p < 0.05); ^□^Statistically different from the eb26 group (p < 0.05). **a** Tb.BMD, **b** BV/TV, **c** Tb.Th, **d** Tb.N, **e** Tb.Sp, **f** Ct.BMD, **g** Ct.Th

The microstructure parameters of trabecular and cortical bones are shown in Fig. 10. No significant differences in Tb.BMD, Ct.BMD and Ct.Th were observed among the groups (p > 0.05). The maximum BV/TV and Tb.Th were noted in the eb12 group; BV/TV was significantly higher in this group than those in the eb19 and eb26 groups (p < 0.05). The lowest BV/TV, Tb.Th, Tb.N and the maximum Tb.Sp were observed in the eb26 group (p < 0.05).

### Nanoindentation test

**Fig. 11:**
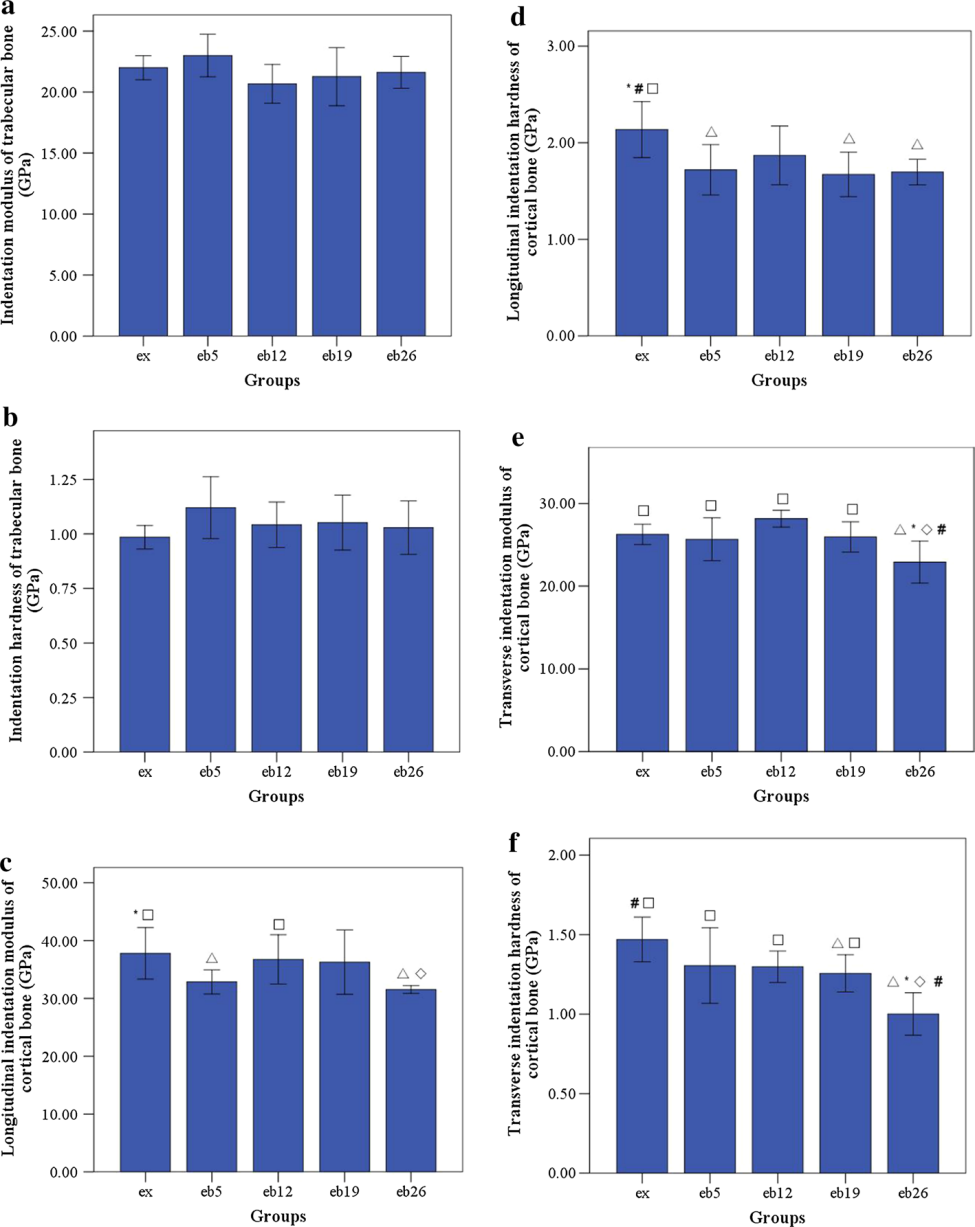
The indentation modulus and hardness of trabecular and cortical bone sites as determined by nanoindentation. Error bars represent SD. *E*_*Tb.L*_ longitudinal indentation modulus of trabecular bone, *H*_*Tb.L*_ longitudinal hardness of trabecular bone, *E*_*Ct.L*_ longitudinal indentation modulus of cortical bone, *H*_*Ct.L*_ longitudinal hardness of cortical bone, *E*_*Ct.T*_ transverse indentation modulus of cortical bone, *H*_*Ct.T*_ transverse hardness of cortical bone. ^△^Statistically different from the ex group (p < 0.05); *Statistically different from the eb5 group (p < 0.05); ^◇^Statistically different from the eb12 group (p < 0.05); ^#^Statistically different from the eb19 group (p < 0.05); ^□^Statistically different from the eb26 group (p < 0.05). **a** *E*_*Tb.L*_ of trabecular bone, **b** *H*_*Tb.L*_ of trabecular bone, **c** *E*_*Ct.L*_ of cortical bone, **d** *H*_*Ct.L*_ of cortical bone, **e** *E*_*Ct.T*_ of cortical bone, **f** *H*_*Ct.T*_ of cortical bone

The indentation moduli and hardness of trabecular and cortical bone sites are shown in Fig. 11. No significant differences in *E*_*Tb.L*_ and *H*_*Tb.L*_ were observed among the groups (p > 0.05).

*E*_*Ct.T*_ and *H*_*Ct.T*_ in the eb26 group were the lowest (p < 0.05); *H*_*Ct.T*_, *H*_*Ct.L*_ in the eb19 group and *E*_*Ct.L*_, *H*_*Ct.L*_ in the eb26 group were significantly lower than that in the ex group (p < 0.05).

## Discussion

This study explored the effects of different weight-bearing levels during treadmill exercise with moderate intensity on the multiscale morphology and the mechanical properties of trabecular and cortical bones in the femur of growing female rats. All rats were subjected to continuous treadmill exercise at moderate intensity with different weight-bearing levels for 8 weeks, the bilateral femurs were evaluated by multiscale methods, and the effects of different weight-bearing levels on bone quality were comprehensively analyzed.

### Effect of weight-bearing during moderate treadmill exercise on trabecular bone

The mechanical properties of trabecular bone structure are related to Tb.BMD [37]. Moderate treadmill exercise with short duration can significantly increase the Tb.BMD of tibia in growing female rats [38]. The BMDs of adult rats are further increased by treadmill exercise with 19% weight-bearing level for 15 min [18]. However, compare with the ex group, increasing weight-bearing exerted no significant increase on the Tb.BMD of proximal femur. The first reason for this result may be that all rats grew rapidly during the experiment, and the body weight also increased; consequently, the effect was impaired due to rapid increase in body weight; second, the sensitivity of bone in growing rats to mechanical stimuli is gender-specific, and the responsiveness of male rats to treadmill exercise is significantly higher than that of female rats [25]. Growing female rats were investigated in the present study. Therefore, the Tb.BMD of femoral head showed no significant increase.

The trabecular bone microstructure is another important factor affecting its mechanical properties [37, 39]. Micro-CT analysis results show that the trabecular bone microstructure was improved significantly in the eb12 group. Nevertheless, similar positive effect was not observed in the eb26 group. This result was consistent with that of our previous study showing that the subchondral bone microstructure in the knee joint of growing female rats is improved by 12% weight-bearing level in combination with treadmill exercise; additionally 26% weight-bearing level causes no positive effect on microstructure [26].

The mechanical properties of trabecular bone are also influenced with its material properties [37, 39]. The indentation moduli and hardness of trabecular bone were obtained by nanoindentation test to characterize its material properties. The *E*_*Tb.L*_ and *H*_*Tb.L*_ showed no significant difference among the groups (Fig. 11). This result demonstrated that the nanomaterial properties of trabecular bone in growing female rats played no significant improvement with increasing weight-bearing during moderate treadmill exercise.

### Effect of weight-bearing during moderate treadmill exercise on cortical bone

Some investigations found that high-intensity treadmill exercise exerts no significant increase in the femoral strength of growing rats [14, 17]. In the present study, although the intensity of treadmill exercise was moderate, the macroelastic moduli of the femur were significantly decreased in the eb19 and eb26 groups. This observation indicated that moderate treadmill exercise in combination with increasing weight-bearing levels exerted no further improvement on the macromechanical properties of the rat femur during the growing period. Considering that mechanostat exists in the bone tissue, if mechanical stimulus is above the adaptive threshold, then bone microdamage and absorption will occur [40–43]. Accordingly, the current result may be related to the excessive weight-bearing level for the growing rats during the experiment.

Ct.BMD is a major factor determining cortical bone strength [37]. Treadmill exercise during growth period significantly increases Ct.BMD in the middle segment of the femur of male rats [22]. Treadmill exercise combined with 19% weight-bearing level in a short period can significantly increase BMD in adult rats compared with that of the treadmill exercise group [18]. Nonetheless, in the present study, the Ct.BMDs were not increased significantly by increasing weight-bearing level during moderate treadmill exercise. The first reason for this result may be that the age and sex of the rats were different. This study used 5-week-old female rats and previous studies used 5-month-old female adult rats and 4-week-old male rats [18, 22]. Moreover, the responses of rat skeleton to mechanical stimulus produced by treadmill exercise are related to their growth stages and sex [20, 25]. Second, the exercise program was different from that in literature. In this study, all rats ran at 12 m/min for 15 min in each day, 7 days/week for 8 weeks, and with 0° slope of the belt; in previous studies, the speed is 20 m/min, the animals run 5 days/week for 17 weeks, and the slope of the belt is 5° [18]. The differences in frequency, intensity and duration of exercise exert different effects on Ct.BMD in rats [1–5].

The nanoscale material property of cortical bone is also an important factor affecting its mechanical properties [44, 45]. The indentation moduli and hardness of cortical bone were obtained by nanoindentation test to characterize its material properties. The *E*_*Ct.T*_, *H*_*Ct.T*_, *E*_*Ct.L*_, and *H*_*Ct.L*_ were not significantly higher than those in the ex group when the weight-bearing levels were increased; instead, 19% and 26% weight-bearing level resulted in a significant decrease in these parameters. This suggested that the nanomechanical properties of cortical bone were not improved significantly by moderate treadmill exercise with weight-bearing during the growth period. Furthermore, the nanomechanical properties may be reduced when the weight-bearing level was higher than 19%.

### Effect of weight-bearing during moderate treadmill exercise on bone formation and resorption

Serum ALP and TRACP are considered ideal bone formation and bone resorption markers [21, 46]. Exercise increases bone mass by increasing the activity of osteoblasts and inhibiting the resorption activity of osteoclasts [3, 24]. In the present study, no significant effect on TRACP was observed for moderate treadmill exercise with increased weight-bearing levels compared with that in the ex group. However, a significant increase in ALP was observed at 5%, 12%, and 19% weight-bearing levels. Thus, increasing weight-bearing levels during moderate treadmill exercise may exert positive effect on bone catabolism.

The present study also displayed several limitations. First, 5-week-old female rats were used in the experiment. Some investigations showed that the responsiveness of bone during growth period to the mechanical stimulus produced by treadmill exercise is gender-specific [25]. In future studies, male rats with the same age should be used to investigate the effects of treadmill exercise with weight-bearing on bone quality and determine the differences in gender response to this type of exercise. Second, no sedentary with weight-bearing groups were set, and the effects of sedentary with weight-bearing activity on bone quality are unknown. Therefore, the sedentary with weight-bearing groups should be added in future study to determine the effects on the bone properties of growing female rats and compare these effects with those of treadmill exercise with weight-bearing. Third, the bone nanomechanical properties are closely related to the contents of Ca and P of cortical bone and its ultrastructure [34, 47–49]. Nevertheless, these cortical bone properties were not investigated. Thus, cortical bone properties at this scale should be assessed in future study to determine their influences on the mechanical properties.

## Conclusions

We confirmed that additional 12% weight-bearing level can significantly increase bone formation, improve microstructure of trabecular bone, and maintain structure and mechanical properties of cortical bone when female rats were treadmill exercised at moderate intensity during the growth period. High weight-bearing level exerted no positive effects on the microstructure of trabecular bone and multiscale properties of cortical bone. Increased weight-bearing levels also exerted no significant influence on body weight, calf muscles weight, femur length, Tb.BMD and Ct.BMD. This study provided a theoretical basis for reasonable exercise among adolescents to improve bone quality.
